# Intercontinental Diversity of *Caballeronia* Gut Symbionts in the Conifer Pest Bug *Leptoglossus occidentalis*

**DOI:** 10.1264/jsme2.ME22042

**Published:** 2022-08-11

**Authors:** Tsubasa Ohbayashi, Raynald Cossard, Gaëlle Lextrait, Takahiro Hosokawa, Vincent Lesieur, Kazutaka Takeshita, Kanako Tago, Peter Mergaert, Yoshitomo Kikuchi

**Affiliations:** 1 Institute for Agro-Environmental Sciences, National Agriculture and Food Research Organization (NARO), 305–8604, Tsukuba, Japan; 2 Université Paris-Saclay, CEA, CNRS, Institute for Integrative Biology of the Cell (I2BC), 91198 Gif-sur-Yvette, France; 3 Department of Biology, Faculty of Science, Kyushu University, 819–0395 Fukuoka, Japan; 4 CSIRO Health and Biosecurity, European Laboratory, Montferrier sur Lez, France; 5 Faculty of Bioresource Sciences, Akita Prefectural University, 010–0195 Akita, Japan; 6 Bioproduction Research Institute, National Institute of Advanced Industrial Science and Technology (AIST), Hokkaido Center, Sapporo 062–8517, Japan; 7 Graduate School of Agriculture, Hokkaido University, Sapporo 060–8589, Japan

**Keywords:** *Caballeronia*, stinkbug, obligate gut symbiosis, intercontinental diversity

## Abstract

Many stinkbugs in the superfamily Coreoidea (Hemiptera: Heteroptera) develop crypts in the posterior midgut, harboring *Caballeronia* (*Burkholderia*) symbionts. These symbionts form a monophyletic group in *Burkholderia sensu lato*, called the “stinkbug-associated beneficial and environmental (SBE)” group, recently reclassified as the new genus *Caballeronia*. SBE symbionts are separated into the subclades SBE-α and SBE-β. Previous studies suggested a regional effect on the symbiont infection pattern; Japanese and American bug species are more likely to be associated with SBE-α, while European bug species are almost exclusively associated with SBE-β. However, since only a few insect species have been investigated, it remains unclear whether region-specific infection is general. We herein investigated *Caballeronia* gut symbionts in diverse Japanese, European, and North American populations of a cosmopolitan species, the Western conifer seed bug *Leptoglossus occidentalis* (Coreoidea: Coreidae). A mole­cular phylogenetic ana­lysis of the 16S rRNA gene demonstrated that SBE-β was the most dominant in all populations. Notably, SBE-α was rarely detected in any region, while a third clade, the “Coreoidea clade” occupied one fourth of the tested populations. Although aposymbiotic bugs showed high mortality, SBE-α- and SBE-β-inoculated insects both showed high survival rates; however, a competition assay demonstrated that SBE-β outcompeted SBE-α in the midgut crypts of *L. occidentalis*. These results strongly suggest that symbiont specificity in the *Leptoglossus*-*Caballeronia* symbiotic association is influenced by the host rather than geography, while the geographic distribution of symbionts may be more important in other bugs.

Recent studies revealed that insect gut microorganisms play a pivotal role in the evolution and environmental adaptation of insects. Gut microorganisms provide essential nutrients, digest indigestible food materials, and/or degrade phytotoxins and insecticides (Kikuchi *et al.*, 2012; Engel and Moran, 2013; Salem *et al.*, 2014; Sudakaran *et al.*, 2017; Itoh *et al.*, 2018; Moran *et al.*, 2019). Many species of stinkbugs in the superfamily Coreoidea (Hemiptera: Heteroptera) develop numerous crypts at the posterior part of the midgut, wherein specific *Caballeronia* symbionts (previously included in the genus *Burkholderia*) densely proliferate - generally as a single species - until almost full occupation of the luminal space (Kikuchi *et al.*, 2005, 2011a; Olivier-Espejel *et al.*, 2011; Garcia *et al.*, 2014; Kuechler *et al.*, 2016; Takeshita and Kikuchi, 2017; Ohbayashi *et al.*, 2019b; Acevedo *et al.*, 2021; Hunter *et al.*, 2022). *Caballeronia* gut symbionts play important roles in their hosts, such as the recycling of metabolic waste materials and providing essential amino acids and vitamins, thereby enhancing the growth and fecundity of stinkbugs (Kikuchi *et al.*, 2007; Kikuchi and Fukatsu, 2014; Ohbayashi *et al.*, 2019a). A particularity of this mono-species symbiosis is the horizontal transmission of symbionts. Hatchlings are symbiont-free and acquire *Caballeronia* symbionts from soil during the early instar stages (Kikuchi *et al.*, 2007, 2011b; Ohbayashi *et al.*, 2019b). This implies not only that insects depend on efficient and stringent selection mechanisms to sort environmental bacteria in order to give access to *Caballeronia* symbionts only (Ohbayashi *et al.*, 2015; Itoh *et al.*, 2019; Kikuchi *et al.*, 2020), but also that the geographic distribution of symbiont species may be a factor influencing the outcome of symbiosis (Ohbayashi *et al.*, 2019b).

The genus *Burkholderia* was initially separated from *Pseudomonas* Group II in 1992 (Yabuuchi *et al.*, 1992) into a heterogeneous taxonomic group of more than 100 bacterial species (Eberl and Vandamme, 2016). In a recent reclassification of this *Burkholderia* “*sensu lato*” taxonomic group, six new genera (*Paraburkholderia*, *Caballeronia*, *Robbsia*, *Mycetohabitans*, *Pararobbsia*, and *Trinickia*) have been proposed next to the genus *Burkholderia sensu strico* (Sawana *et al.*, 2014; Dobritsa and Samadpour, 2016; Beukes *et al.*, 2017; Lopes-Santos *et al.*, 2017; Estrada-de Los Santos *et al.*, 2018; Lin *et al.*, 2020). *Caballeronia* is also called the Stinkbug-associated Beneficial and Environmental (SBE) group of *Burkholderia*, which is divided into two subgroups, group-α (SBE-α) and group-β (SBE-β).

A previous survey of *Caballeronia* symbionts in stinkbugs revealed region-specific infection with species of the two subgroups: SBE-α was more likely to be detected in Japanese and American stinkbug species of Coreoidea (Kikuchi *et al.*, 2005, 2011b; Olivier-Espejel *et al.*, 2011; Garcia *et al.*, 2014; Kuechler *et al.*, 2016; Ohbayashi *et al.*, 2019b; Acevedo *et al.*, 2021; Hunter *et al.*, 2022), and SBE-β in European species of Coreoidea (Kuechler *et al.*, 2016; Ohbayashi *et al.*, 2019b). However, since only a few insect species have been investigated, and even fewer species from a wide geographic distribution, it remains unclear whether region-specific infection is general. The mechanisms underlying region-specific infection have not yet been elucidated, but may be influenced by the region-dependent composition of the soil microbiota.

The Western conifer seed bug *Leptoglossus occidentalis* (Coreoidea: Coreidae) (Fig. 1A), a notorious pest of conifer forests (Lesieur *et al.*, 2014), originates from North America (Heidemann, 1910; Koerber, 1963). This stinkbug has become a serious invading pest worldwide. In 1999, *L. occidentalis* was found in Europe for the first time in Italy (Taylor *et al.*, 2001) and its population has been rapidly expanding in recent years throughout Europe (Dusoulier *et al.*, 2007; Malumphy *et al.*, 2008; Fent and Kment, 2011; Gapon, 2013; Lesieur *et al.*, 2018; van der Heyden, 2019) and other more distant regions, such as North Africa (Ben Jamaa *et al.*, 2013; Gapon, 2015). It has also spread to South America (Faúndez *et al.*, 2017) and Asia (Ishikawa and Kikuhara, 2009; Ahn *et al.*, 2013). In Japan, *L. occidentalis* was initially collected in Tokyo in 2008 (Ishikawa and Kikuhara, 2009) and, similar to Europe, it has since rapidly spread to almost all areas of Japan, including the Tohoku and Kyushu districts (Tsuru *et al.*, 2020). Therefore, this cosmopolitan species is ideal for clarifying regional effects on the symbiotic association. In the present study, we investigated the diversity of *Caballeronia* symbionts of *L. occidentalis* collected in Japan, North America, and Europe in order to confirm whether the geographical origin affects the gut symbionts of the conifer bug. Furthermore, symbiont inoculation tests of insects reared in the laboratory with SBE-α and SBE-β symbionts were conducted to analyze the gut colonization ability and fitness effects of these two *Caballeronia* subgroups on the host insect.

## Materials and Methods

### Insects

Samples of *L. occidentalis* used in the present study are listed in Table 1. Regarding bacterial inoculation tests, *L. occidentalis* was collected in Gif-sur-Yvette, France in 2018 and maintained in cages by feeding on pignolia nuts and distilled water containing 0.05% ascorbic acid (DWA) at 25°C under a long-day regime (16 h light, 8 h dark).

### Symbiont inoculation tests

Reared insects were used for inoculation tests with one SBE-α strain and one SBE-β strain. We used strain RPE225 (Kikuchi and Fukatsu, 2014), a green fluorescent protein (GFP)-labeled derivative of *B. insecticola *(*Caballeronia insecticola*) strain RPE64, as a typical strain of the SBE-α clade. RPE64 was isolated from the midgut crypts of a Japanese specimen of the bean bug *Riptortus pedestris* (Coreoidea: Alydidae) (Takeshita *et al.*, 2018). Regarding the SBE-β strain, we selected *Caballeronia* sp. strain 1876, which was isolated from the midgut crypts of *L. occidentalis* collected in Gif-sur-Yvette, France in 2016. A GFP-labeled derivative of this SBE-β strain, labeled strain 2482, was constructed by a mini-Tn7 transposon delivery system as previously reported (Kikuchi and Fukatsu, 2014). Insect inoculation tests with these two GFP strains were performed as previously described for other stinkbug species (Ohbayashi *et al.*, 2015; Ohbayashi *et al.*, 2019b). Briefly, the two bacterial strains were pre-cultured in yeast extract and glucose (YG) medium (yeast extract 5.0‍ ‍g L^–1^, glucose 4.0‍ ‍g L^–1^, and NaCl 1.0‍ ‍g L^–1^) containing rifampicin 30‍ ‍μg mL^–1^ at 28°C overnight at 180‍ ‍rpm, and 200‍ ‍μL of the overnight culture was inoculated into 5‍ ‍mL fresh YG, incubated at 28°C at 180‍ ‍rpm for 2 h, and finally diluted to 10^7^ CFU mL^–1^ in DWA. The cotton pad with DWA was removed from the rearing container with 2^nd^ instar *L. occidentalis* nymphs, and nymphs were maintained overnight without water to make them thirsty. Symbiont suspensions, prepared as described above, were poured onto new cotton pads and placed into rearing containers. Aposymbiotic nymphs were obtained by placing a cotton pad soaked with DWA only. Containers were maintained as described above until later ana­lyses.

### Fluorescence microscopy observations

The infection status of the inoculated nymphs was confirmed based on the detection of GFP signals in the dissected midgut crypts of third instar nymphs. Dissections were performed in phosphate-buffered saline (PBS) using fine forceps and scissors under a fluorescent binocular microscope (Leica, MZ FZ III). Pictures of the dissected midguts were taken by a digital camera (Leica, EC3).

### Insect survival

The survival rate of aposymbiotic insects and symbiotic insects infected with either SBE-α strain RPE225 or SBE-β strain 2482 was estimated by regularly observing insect rearing populations (*n*=27, 13 or 20 insects, respectively) over time until the last adult emergence in the surviving insects. At each observation, the number of alive and dead insects was scored, as well as the number of emerged adults. The survival rate was analyzed by Fisher’s exact test with the Bonferroni correction. The developmental time until adulthood in the aposymbiotic insect sample was removed from the statistical ana­lysis due to a single surviving insect (*n*=1), and those in SBE-α and SBE-β inoculated insects were analyzed by the Student’s *t*-test.

### Competition assay

In the competition assay, we used red fluorescent protein (RFP) strain RPE525 (SBE-α), a derivative of *C. insecticola* strain RPE64 (Itoh *et al.*, 2019) and GFP strain 2482 (SBE-β). Exponential phase cells were suspended in DWA and an inoculum containing 10^7^ CFU mL^–1^ of both strains was prepared from them. The inoculation of insects with the mixed inoculum was performed as described above. At 7 days post inoculation, when insects became 3^rd^ instar nymphs, midgut crypts were dissected as described above. In the microscopy ana­lysis, midgut crypts were observed under a fluorescent microscope (Nikon, Eclipse 80i). Regarding the quantitative assessment of the two strains, midgut crypts were collected in 100‍ ‍μL of PBS buffer in a 1.5-mL tube and homogenized by a sterilized pestle. The pestle was washed by 400‍ ‍μL of PBS buffer. The relative number of symbiont cells of GFP and RFP bacteria in the extracts of the midgut crypts and in the bacterial suspension of the inoculum were analyzed by flow cytometry (Beckman Coulter, Cytoflex).

### Identification of gut symbionts of *L. occidentalis*

**Fig. 1.**
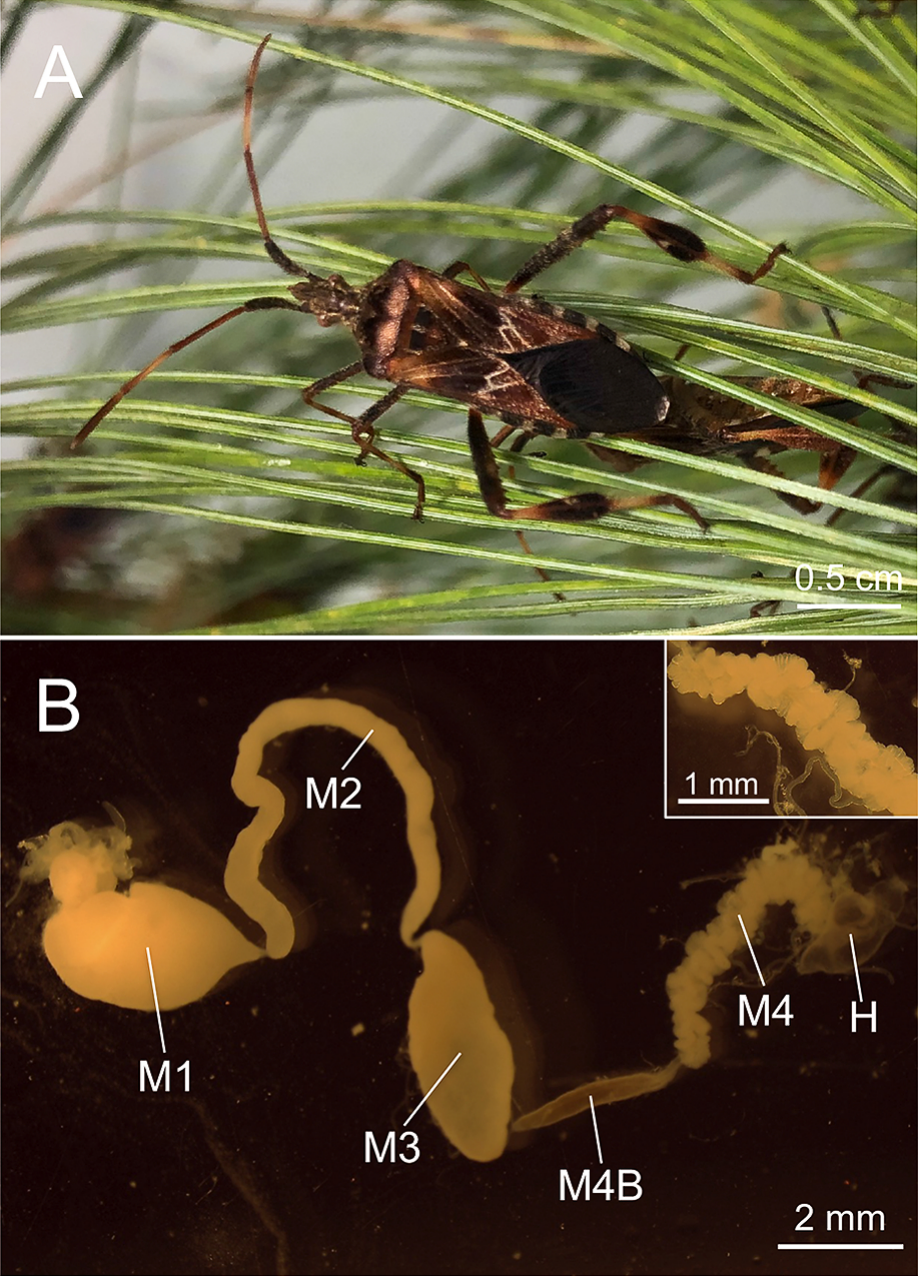
The conifer bug *Leptoglossus occidentalis* and its midgut structure. (A) An adult of *L. occidentalis* on the leaves of a pine tree and (B) its whole midgut structure. The bug shown was reared in the laboratory. Symbiont inoculation was performed by feeding the insect a soil suspension. A symbiont native in the soil colonizes the midgut crypts (M4). Abbreviations of the midgut sections are as follows: M1, midgut first section; M2, midgut second section; M3, midgut third section; M4, midgut fourth section with crypts; M4B, M4 bulb; H, hindgut.

Gut symbionts were isolated from the midgut crypts of *L. occidentalis* individuals collected in Japan and France (Table 1 and Fig. 1) by plating crypt contents on YG agar plates. Growing bacteria were identified by direct sequencing of the 16S rRNA gene, as previously described (Kikuchi *et al.*, 2011a). Since conifer bugs captured in Italy, Spain, USA, and Canada were preserved in 100% ethanol, their dissected midgut crypts were subjected to DNA extraction and a clone library ana­lysis of the 16S rRNA gene, as previously reported (Ohbayashi *et al.*, 2019b). Sequences obtained by the bacterial isolation and clone ana­lysis were assembled by ATSQ software ver. 5.2 (Software Development), followed by manual corrections. The most similar bacterial species/strains were identified by BLAST comparisons. Sequences showing more than 99% identity were assigned to the same operational taxonomical unit (OTU).

### Molecular phylogenetic ana­lysis of *L. occidentalis* gut symbionts

A multiple sequence alignment of the 16S rRNA gene was constructed with MAFFT on the EMBL-EBI server (Li *et al.*, 2015). A mole­cular phylogenetic tree was generated by the maximum likelihood (ML) method with the removal of gap-including and ambiguous sites and with a bootstrap ana­lysis (1,000 replicates) in MEGA software version 10.1.8 (Kumar *et al.*, 2018; Stecher *et al.*, 2020). We selected the Tamura-Nei model of nucleotide substitutions with gamma distributed and invariant sites (G+I) (Tamura and Nei, 1993).

### Nucleotide sequence accession numbers

The nucleotide sequence data of the 16S rRNA gene obtained in the present study have been deposited in the DDBJ/EMBL/GenBank public databases with the accession numbers LC713090–LC713209 (Table 1).

## Results

### Identification of gut symbionts in midgut crypts of *L. occidentalis*

To investigate the diversity of gut symbionts in the conifer bug *L. occidentalis*, two methods were used depending on the nature of the insect sample. In the bacterial isolation method, 43 symbionts were isolated from the midgut crypts of 22 individuals collected from 4 and 2 populations of Japan and France, respectively (Table 1). In the clone library ana­lysis of the 16S rRNA gene, 17 specimens collected in Italy, Spain, USA, and Canada were used, from which 77 sequences were obtained (Table 1). The 120 assembled sequences were assigned to 11 OTUs based‍ ‍on the‍ ‍99% sequence identity threshold (Table S1). Seven OTUs (OTU1–OTU7) were identified as members of *Caballeronia* by a BLAST search. OTU2 and OTU7 represented 69% and 22%, respectively, of the 110 sequences identified as *Caballeronia*, indicating that OTU2 and OTU7 are the main gut symbionts of *L. occidentalis*. These dominant OTUs were detected in both the bacterial isolation and cloning methods, which suggests no method-related bias in the identification of symbionts. The remaining four OTUs (OTU8–OTU11), detected only in four individuals of two insect populations, were identified as *Rickettsia* spp., which is a well-known intracellular secondary symbiont of diverse insects (Kikuchi, 2009). The insect specimen in which *Rickettsia* clones were identified, yielded a majority of *Caballeronia* clones (Table S1), indicating that these individuals were also colonized with *Caballeronia* symbionts.

### Phylogenetic placement of *Caballeronia* symbionts

**Fig. 2.**
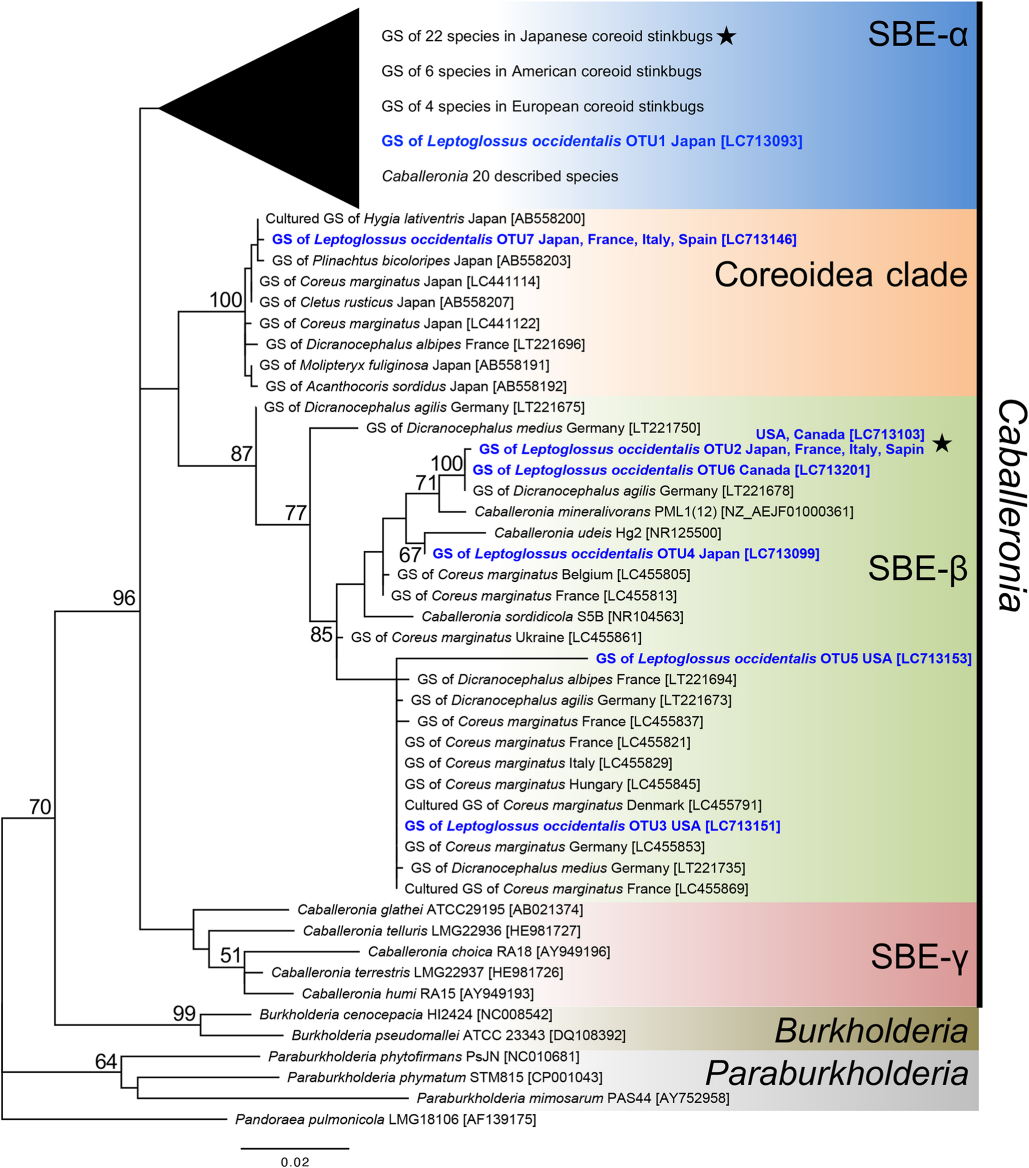
Molecular phylogenetic ana­lysis of *Caballeronia* gut symbionts of the conifer bug* Leptoglossus occidentalis*. A maximum-likelihood tree was generated based on 1,256 aligned nucleotide sites of the 16S rRNA gene. Numbers at the tree nodes indicate the maximum-likelihood bootstrap values (%) with 1,000 replicates, and bootstrap values of more than 50 are shown. We referred to the nucleotide sequence information reported in previous studies on the *Caballeronia* gut symbionts of coreoid insects in Japan (Kikuchi *et al.*, 2011a; Kuechler *et al.*, 2016; Ohbayashi *et al.*, 2019b), in America (Olivier-Espejel *et al.*, 2011; Garcia *et al.*, 2014; Acevedo *et al.*, 2021; Hunter *et al.*, 2022), and in Europe (Kuechler *et al.*, 2016; Ohbayashi *et al.*, 2019b). The subtree of the SBE-α group is compressed. An uncompressed subtree is shown in Fig. S1. Accession numbers in the DNA database (DDBJ/EMBL/GenBank) are shown in square brackets. *L. occidentalis* gut symbionts are shown in blue with bold case font. Stars: bacterial strains used for symbiont inoculation tests. GS: Gut symbiont.

We performed a mole­cular phylogenetic ana­lysis based on the 16S rRNA gene, including sequences of the seven *Caballeronia* OTUs of *L. occidentalis* gut symbionts, type strains of *Burkholderia sensu lato* species (*Burkholderia sensu strico*, *Paraburkholderia*, and *Caballeronia* species), and previously reported *Caballeronia* gut symbionts of various coreoid insects. Based on phylogenetic divergence, four subclades were identified within the *Caballeronia* clade: SBE-α, SBE-β, SBE-γ, and Coreoidea clade (Fig. 2). The most dominant OTU2, detected in specimens collected in all countries, was located in SBE-β. SBE-β contained four other OTUs (OTU3, OTU4, OTU5, and OTU6) in addition to OTU2 (Fig. 2). On the other hand, the second most dominant OTU7 was placed in the Coreoidea clade (Fig. 2). This subclade contained neither environmental isolates nor type species, but formed a monophyletic group with many gut symbionts of Japanese and European coreoid bugs (Fig. 2), suggesting their very specialized nature for symbiosis with stinkbug species. OTU1 was located in the SBE-α clade, known as major gut symbionts of Japanese and American coreoid stinkbugs (Fig. 2 and S1).

**Fig. 3.**
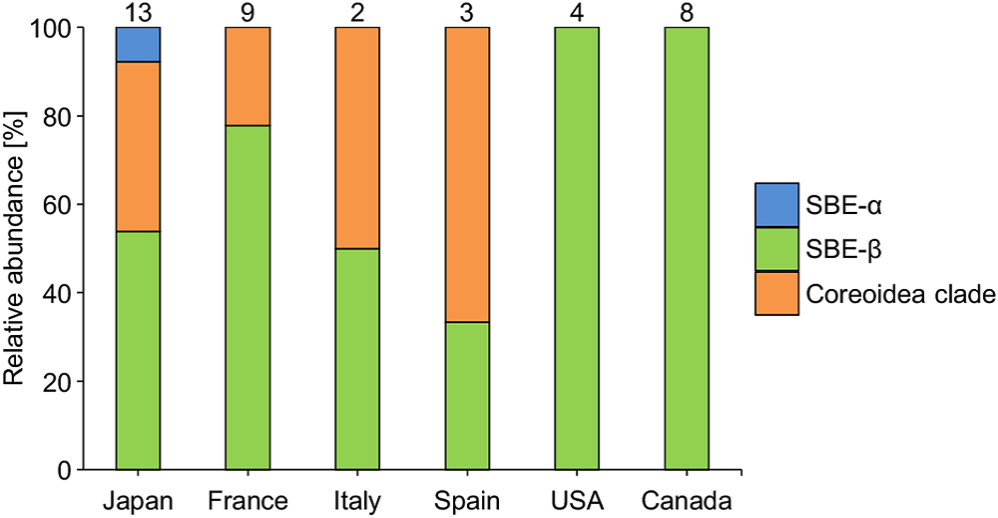
Relative abundance of SBE-α, SBE-β, and Coreoidea clade bacteria among gut symbionts of conifer bugs normalized by one OTU by an individual at the country level. The number of investigated insects in each country is shown in the graphs, and the precise numbers are provided in Table S1 and S2. Relative abundance at the local level is shown in Fig. S2.

The detection rates of the three subclades (SBE-α, SBE-β, and the Coreoidea clade) in the world’s populations of conifer bugs are summarized in Fig. 3 and S2 (also see Table S2), which are based on the phylogenetic placement of seven OTUs in the subclades of *Caballeronia* (Fig. 2). Overall, conifer bugs were almost exclusively associated with SBE-β, while SBE-α was rarely detected (Fig. 3). The Coreoidea clade occupied one fourth of the populations and was frequently detected in the Japanese and European populations, but not in the North American populations of conifer bugs (Fig. 3).

### Colonization ability and host fitness effects of SBE-α and SBE-β symbiont strains in the midgut crypts of *L. occidentalis*

**Fig. 4.**
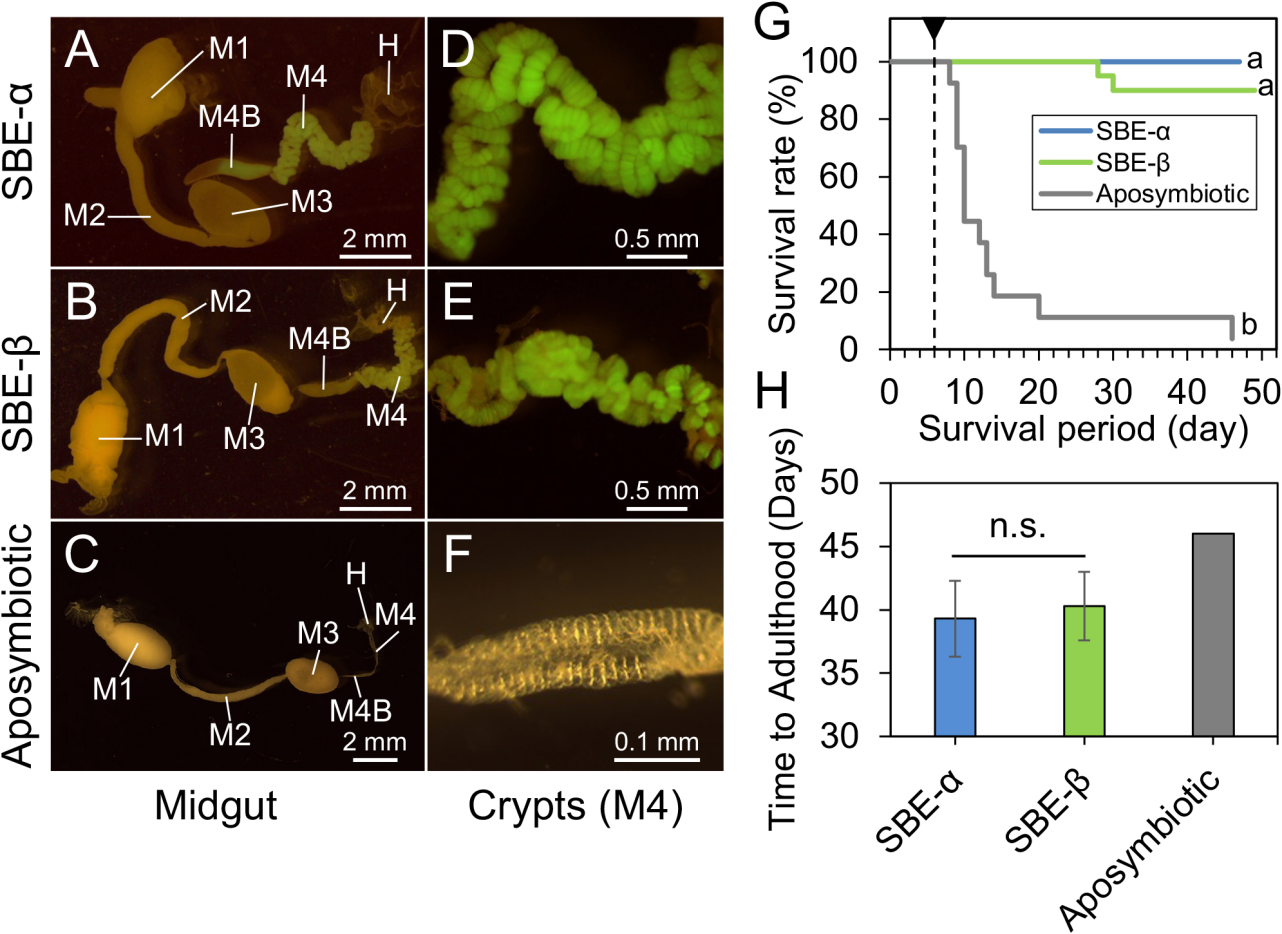
*Leptoglossus occidentalis* infection with SBE-α and SBE-β strains and their host fitness effects. (A, B, and C) Whole midguts of *L. occidentalis* at the 3^rd^ instar stage, and (D, E, and F) an enlarged image of midgut crypts. A dissected midgut inoculated with (A and D) an SBE-α GFP strain, with (B and E) an SBE-β GFP strain, and without (C and F) any inoculant (aposymbiotic). An abbreviation of the midgut section is as shown in Fig. 1. (G) Survival rates of *L. occidentalis* inoculated with SBE-α (blue line, *n*=13) and SBE-β (green, *n*=20), and without any inoculant (aposymbiotic: gray, *n*=27). The survival period was followed from hatching to the last adult emergence. A black arrow and dotted line indicate symbiont infections at 6 days post-hatching. Different letters indicate significant differences (*P*<0.0001, Fisher’s exact test with the Bonferroni correction). (H) Developmental time from hatching to adulthood in conifer bugs inoculated with SBE-α (blue bar, *n*=13) and SBE-β (green, *n*=18), and without any inoculant (aposymbiotic: gray line, *n*=1, *i.e.* the only surviving insect in the experiment). The mean±SD is shown. n.s. indicates no significant difference (Student’s *t*-test).

SBE-α species have consistently been found in the midgut crypts of 33 stinkbug species of the superfamily Coreoidea (Kikuchi *et al.*, 2011a, 2005; Olivier-Espejel *et al.*, 2011; Garcia *et al.*, 2014; Kuechler *et al.*, 2016; Ohbayashi *et al.*, 2019b; Acevedo *et al.*, 2021; Hunter *et al.*, 2022). However, in the present study, only one out of 120 symbiont isolates or clones from 39 specimens of *L. occidentalis* were SBE-α (Fig. 3 and Table S1). To confirm whether SBE-α and SBE-β symbionts are capable of colonizing the midgut crypts of the conifer bug, the GFP-labeled strains, *C. insecticola* strain RPE225 (SBE-α) and *Caballeronia* sp. strain 2482 (SBE-β) were inoculated into nymphs of *L. occidentalis*. The SBE-α and SBE-β strains were both capable of colonizing the midgut crypts of *L. occidentalis* (Fig. 4A, B, D, and E), as indicated by enlarged crypts and the presence of a GFP signal. Moreover, the swollen M4B midgut region, typical for symbiont colonization, also confirmed proper colonization by both strains (Fig. 4A and B). On the other hand, aposymbiotic (uninfected) insects showed small M4 crypts and a narrow M4B region (Fig. 4C and F).

We then investigated the survival of aposymbiotic and SBE-α- or SBE-β-inoculated conifer bugs. Aposymbiotic bugs showed high mortality during their development (Fig. 4G). The majority of insects died during the 2^nd^ to 3^rd^ instar (Fig. 4G). In contrast, most nymphs inoculated with SBE-α or SBE-β strains survived and reached adulthood (Fig. 4G; survival percentage [adult/total investigated insects]=3.7% [1/27] in aposymbiotic insects; 100% [13/13] in SBE-α-inoculated insects; 90% [18/20] in SBE-β-inoculated insects). The developmental times from hatching to adult emergence were 46, 39±3, and 40±3 days (mean±SD) in aposymbiotic, SBE-α-, and SBE-β-inoculated *L. occidentalis*, respectively (Fig. 4H). Survival rates significantly differed between symbiotic (inoculated with SBE-α or SBE-β) versus aposymbiotic insects, while no significant differences were observed in survival rates or developmental times between SBE-α- and SBE-β-inoculated *L. occidentalis* (Fig. 4G and H).

**Fig. 5.**
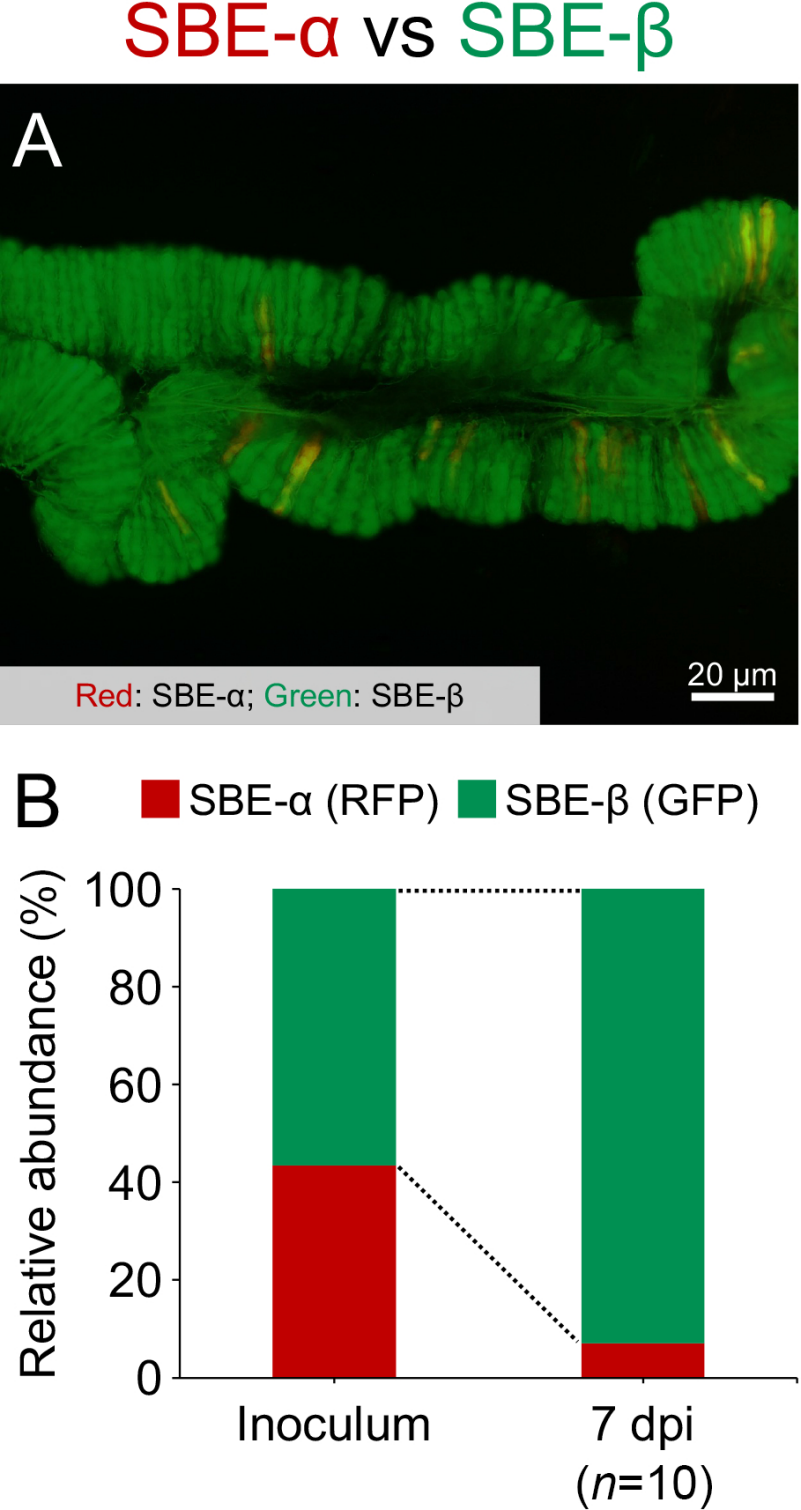
Competition assay of RFP-labeled SBE-α and GFP-labeled SBE-β strains in midgut crypts of *Leptoglossus occidentalis*. (A) The midgut crypts of *L. occidentalis* at 7 days post-infection (dpi), infected with an equal mixture of both strains. A merged GFP and‍ ‍RFP image is shown. (B) Relative abundance, determined by flow cytometry, of GFP and RFP strains at the inoculum and at the midgut crypts at 7 dpi (*n*=10). The abundance of GFP and RFP strains at the midgut crypts is significantly different (*P*<1×10^–10^, the Student’s *t*-test). Fluorescent microscopic images and the relative abundance of GFP and RFP strains in 10 individual insects are shown in Fig. S3. Note that both strains resulted in 100% infection when used in control mono-infections (Fig. S4).

On the other hand, a competition assay, in which nymphs were inoculated with an equal mixture of the SBE-α and SBE-β strains, demonstrated that SBE-β significantly outcompeted SBE-α in the midgut crypts of *L. occidentalis* (Fig. 5). Collectively, these results strongly suggest that the *Caballeronia* symbiont makes a very large positive contribution to the survival and development of *L. occidentalis*, and that SBE-α has a sufficient ability to colonize midgut crypts to give the same fitness effects to *L. occidentalis* as SBE-β. Nevertheless, microbe-microbe competition in the midgut crypts of *L. occidentalis* may contribute to the observed predominance of SBE-β.

## Discussion

The present study revealed that although conifer bug specimens are associated with genetically diverse *Caballeronia* (SBE-*Burkholderia*) symbionts, members of the subclade SBE-β were dominant in all of the investigated Japanese, European, and North American populations of the conifer bug, and the Coreoidea clade was also frequently found in Japanese and European insects (Fig. 2 and 3, Table S1 and S2). Previous studies reported that Japanese and American species of the stinkbug superfamily Coreoidea were more likely to harbor symbionts belonging to the SBE-α subclade (Kikuchi *et al.*, 2011a; Ohbayashi *et al.*, 2019b); however, we herein demonstrated that SBE-α was rarely detected in the conifer bug, even in Japanese and American populations (Fig. 3, Table S1 and S2). Based on this broad survey of this cosmopolitan species, we concluded that infection was not affected by geographic origins; therefore, it is more likely to be influenced by selection mechanisms in the host insect.

Experimental inoculation tests revealed no significant differences in colonization ability or fitness effects on the host bug between SBE-α and SBE-β symbionts (Fig. 4). However, the competition assay showed that SBE-β outcompeted SBE-α in the midgut crypts of *L. occidentalis*, possibly resulting in the low detection rate of SBE-α in the conifer bug (Fig. 3, Table S1 and S2). Infection specificity between the bean bug host and *Caballeronia* symbiont was demonstrated to be largely influenced by the native symbiont’s colonization competitiveness in the midgut. In midgut crypts in co-infection experiments, *Caballeronia* symbionts always outcompeted the non-symbiont species *Paraburkholderia* and *Pandoraea*, which were fully capable of colonizing crypts in the absence of competition species (Itoh *et al.*, 2019). The present study is the first to report competition-based selection in the stinkbug midgut between species of different SBE clades. Since competition-based mechanisms have not yet been elucidated in detail, further studies are warranted.

Among the *Caballeronia* gut symbionts of *L. occidentalis*, the second most dominant OTU7 formed a monophyletic group, the Coreoidea clade, with gut symbionts of other Coreoid species, including *Cletus rusticus*, *Plinachtus bicoloripes*, *Hygia lativentris*, *Molipteryx fuliginosa*, *Acanthocoris sordidus* (Kikuchi *et al.*, 2011a), *Coreus marginatus* (Ohbayashi *et al.*, 2019b), and *Dicranocephalus albipes* (Kuechler *et al.*, 2016). The Coreoidea clade does not include environmental isolates/clones or any named species of *Caballeronia*, it only consists of gut symbionts of Coreoidea (Fig. 2), strongly suggesting that these symbiont strains are highly specific to the insect group. To date, two strains—one from *A. sordidus* and the other the OTU7 clone from *L. occidentalis* described herein—have been isolated as culturable symbionts of this clade. Further studies are needed to reveal the future genomic and physiological features of these Coreoidea clade symbionts in order to clarify why these Coreoidea clade members are specific to this insect group. The intercontinental infection pattern of the Coreoidea clade (Fig. 3) is notable from the viewpoint of evolution. Since *L. occidentalis* originated from North America (Heidemann, 1910; Koerber, 1963) and recently invaded European and Asian countries, we speculate that *L. occidentalis* was originally associated with SBE-β and may have become symbiotic with the Coreoidea clade as its distribution expanded.

*Caballeronia* symbionts make a very large positive contribution to the fitness of the conifer bug (Fig. 4G), similar to that reported for other coreoid stinkbugs, including *C. marginatus*, *L. zonatus*, *L. phyllopus*, and *R. pedestris* (Kikuchi *et al.*, 2007, 2011a; Ohbayashi *et al.*, 2019b; Hunter *et al.*, 2022). In *R. pedestris*, our previous transcriptomic study revealed that *Caballeronia* provided the host with essential amino acids and vitamins by recycling host metabolic waste materials, such as sulfate, allantoin, and urea (Ohbayashi *et al.*, 2019a). These findings suggest that *Caballeronia* symbionts critically complement these essential nutrients lacking in conifer seeds in *L. occidentalis*, resulting in high mortality in aposymbiotic nymphs in *L. occidentalis*. In contrast, aposymbiotic insects in *R. pedestris* showed retarded growth, a smaller body size,‍ ‍and lower fecundity than symbiotic insects, although these aposymbiotic nymphs were nevertheless able to develop to adulthood with a high survival rate (Kikuchi *et al.*, 2007; Kikuchi and Fukatsu, 2014). Feeding on soybean seeds with high nutritional value may provide sufficient, albeit non-optimal, nutrition for the development and survival of aposymbiotic *R. pedestris*. *Caballeronia* symbionts may play more important metabolic roles for hosts that are feeding on nutritionally poor non-leguminous plants, such as *L. occidentalis*.

*Rickettsia* was detected in the American and Canadian populations of *L. occidentalis* (Table S1). Although some members are human pathogens, most members of the genus *Rickettsia* are facultative intracellular symbionts in many arthropods (Perlman *et al.*, 2006), and these symbionts are maintained by vertical transmission. Since *Rickettsia* is a reproductive manipulator that causes male-killing and parthenogenesis in many insects (Perlman *et al.*, 2006), a similar function needs to be considered in the North America populations of the conifer seed bug. The bacterial group has rarely been detected from stinkbug species, except for some species of Miridae (Chang and Musgrave, 1970; Caspi-Fluger *et al.*, 2012; Dally *et al.*, 2020), and a broader survey is required to clarify the prevalence of *Rickettsia* in heteropteran insects.

The present study on *L. occidentalis* is the first to demonstrate that the specificity of the *Caballeronia* symbiont is influenced by host species rather than biogeography. The symbiont’s competitiveness in the gut symbiotic organ appears to be pivotal for specificity. Additionally, differences in fitness effects on host bugs between species, as shown in* L. zonatus* and *L. phyllopus* (Hunter *et al.*, 2022), may be involved in specificity. To elucidate the mechanisms underlying host-symbiont specificity in stinkbugs in more detail, the following two points need to be clarified in the future. The worldwide distribution of *Caballeronia* in soil needs to be analyzed because microbial geography is critical in animals that are closely associated with environmentally transmitted beneficial microorganisms. In addition, further experimental inoculation assays, particularly *in vivo* competition assays, are crucial for confirming and understanding the mechanisms underlying host-microbe specificity in each stinkbug species.

## Citation

Ohbayashi, T., Cossard, R., Lextrait, G., Hosokawa, T., Lesieur, V., Takeshita, K., et al. (2022) Intercontinental Diversity of *Caballeronia* Gut Symbionts in the Conifer Pest Bug *Leptoglossus occidentalis*. *Microbes Environ ***37**: ME22042.

https://doi.org/10.1264/jsme2.ME22042

## Supplementary Material

Supplementary Material

## Figures and Tables

**Table 1.** Information on insect samples investigated in this study.

Country	State/Prefecture	Locality	Collection year	Collector	Symbiont detection	Specimens number	Symbiont isolates/ clones number	16S rRNA Accession number
Japan	Kumamoto	Koshi	March, 2021	R. Hara, K. Matsunaga	Isolating	3	3	LC713090–LC713092
	Yamagata	Yuza	April, 2021	Y. Hatanaka	Isolating	4	4	LC713093–LC713096
	Akita	Akita	October, 2018	K. Takeshita	Isolating	2	2	LC713097–LC713098
	Akita	Akita	April, 2021	S. Noriyuki	Isolating	4	4	LC713099–LC713102
France	Essonne	Gif-sur-Yvette^a^	November, 2016	P. Mergaert	Isolating	3	8	LC713103–LC713110
	Essonne	Gif-sur-Yvette^a^	November, 2021	G. Lextrait	Isolating	6	22	LC713111–LC713132
Italy	Piedmont	Alessandria	October 2020	S. Chiesa	Cloning	2	6	LC713133–LC713138
Spain	Catalonia	Artes	April 2021	S. Lopez Romero	Cloning	3	12	LC713139–LC713150
USA	Idaho	Lenore	October 2020	S. Cook	Cloning	4	31	LC713151–LC713181
Canada	Nova Scotia	Vaughan	October 2020	S. Blatt	Cloning	4	13	LC713182–LC713194
	British Col.	Vernon	October 2020	W. Strong	Cloning	4	15	LC713195–LC713209

^a^ The same collection site
