# Supplementary material for: Intercontinental Diversity of Caballeronia Gut Symbionts in the Conifer Pest Bug Leptoglossus occidentalis
Source: Microbes Environ. 2022 Aug 11;37(3):ME22042. doi: 10.1264/jsme2.ME22042 (PMC9530724; doi:10.1264/jsme2.ME22042)
Supplement: Supplementary file 1 — Supplementary Material [file 37_22042_s1.pdf]

Table S1. OTUs profile of the 16S rRNA gene in *Caballeronia* gut symbionts and *Rickettsia* of *Leptoglossus occidentalis*

Symbiont detection	Collection location		Individual No.	SBE-α	SBE-β					Coreoidea-clade	Rickettsia				Total
				OTU1	OTU2	OTU3	OTU4	OTU5	OTU6	OTU7	OTU8	OTU9	OTU10	OTU11	
Bacterial isolation	Japan	Kumamoto	#1	–	–	–	–	–	–	1	–	–	–	–	1
			#2	–	–	–	–	–	–	1	–	–	–	–	1
			#3	–	–	–	–	–	–	1	–	–	–	–	1
		Yamagata	#1	1	–	–	–	–	–	–	–	–	–	–	1
			#2	–	1	–	–	–	–	–	–	–	–	–	1
			#3	–	–	–	–	–	–	1	–	–	–	–	1
			#4	–	1	–	–	–	–	–	–	–	–	–	1
		Akita, 2016	#1	–	–	–	1	–	–	–	–	–	–	–	1
			#2	–	–	–	1	–	–	–	–	–	–	–	1
		Akita, 2021	#1	–	–	–	1	–	–	–	–	–	–	–	1
			#2	–	–	–	–	–	–	1	–	–	–	–	1
			#3	–	1	–	–	–	–	–	–	–	–	–	1
			#4	–	1	–	–	–	–	–	–	–	–	–	1
Bacterial isolation	France	Gif, 2016	#1	–	2	–	–	–	–	–	–	–	–	–	2
			#2	–	3	–	–	–	–	–	–	–	–	–	3
			#3	–	3	–	–	–	–	–	–	–	–	–	3
		Gif, 2021	#1	–	2	–	–	–	–	–	–	–	–	–	2
			#2	–	4	–	–	–	–	–	–	–	–	–	4
			#3	–	–	–	–	–	–	4	–	–	–	–	4
			#4	–	–	–	–	–	–	4	–	–	–	–	4
			#5	–	4	–	–	–	–	–	–	–	–	–	4
			#6	–	4	–	–	–	–	–	–	–	–	–	4
Cloning	Italy	Alessandria	#1	–	–	–	–	–	–	3	–	–	–	–	3
			#3	–	3	–	–	–	–	–	–	–	–	–	3
Cloning	Spain	Artes	#1	–	4	–	–	–	–	–	–	–	–	–	4
			#2	–	–	–	–	–	–	3	–	–	–	–	3
			#3	–	–	–	–	–	–	5	–	–	–	–	5
Cloning	USA	Lenore	#1	–	–	4	–	1	–	–	3	–	–	1	9
			#2	–	5	–	–	–	–	–	1	–	1	–	7
			#3	–	9	–	–	–	–	–	2	1	–	–	12
			#4	–	3	–	–	–	–	–	–	–	–	–	3
Cloning	Canada	Vaughan	#1	–	3	–	–	–	–	–	–	–	–	–	3
			#2	–	3	–	–	–	–	–	–	–	–	–	3
			#3	–	3	–	–	–	–	–	1	–	–	–	4
			#4	–	3	–	–	–	–	–	–	–	–	–	3
		Vernon	#1	–	3	–	–	–	–	–	–	–	–	–	3
			#2	–	3	–	–	–	1	–	–	–	–	–	4
			#3	–	5	–	–	–	–	–	–	–	–	–	5
			#4	–	3	–	–	–	–	–	–	–	–	–	3
			Total	1	76	4	3	1	1	24	7	1	1	1	120

Table S2. OTUs profile of the 16S rRNA gene in *Caballeronia* gut symbionts of *Leptoglossus occidentalis* normalized by one OTU per one individual host.

Symbiont detection	Collection location		Individual No.	SBE-α	SBE-β					Coreoidea-clade	Total
				OTU1	OTU2	OTU3	OTU4	OTU5	OTU6	OTU7	
Bacterial isolation	Japan	Kumamoto	#1	–	–	–	–	–	–	1	1
			#2	–	–	–	–	–	–	1	1
			#3	–	–	–	–	–	–	1	1
		Yamagata	#1	1	–	–	–	–	–	–	1
			#2	–	1	–	–	–	–	–	1
			#3	–	–	–	–	–	–	1	1
			#4	–	1	–	–	–	–	–	1
		Akita, 2016	#1	–	–	–	1	–	–	–	1
			#2	–	–	–	1	–	–	–	1
		Akita, 2021	#1	–	–	–	1	–	–	–	1
			#2	–	–	–	–	–	–	1	1
			#3	–	1	–	–	–	–	–	1
			#4	–	1	–	–	–	–	–	1
Bacterial isolation	France	Gif, 2016	#1	–	1	–	–	–	–	–	1
			#2	–	1	–	–	–	–	–	1
			#3	–	1	–	–	–	–	–	1
		Gif, 2021	#1	–	1	–	–	–	–	–	1
			#2	–	1	–	–	–	–	–	1
			#3	–	–	–	–	–	–	1	1
			#4	–	–	–	–	–	–	1	1
			#5	–	1	–	–	–	–	–	1
			#6	–	1	–	–	–	–	–	1
Cloning	Italy	Alessandria	#1	–	–	–	–	–	–	1	1
			#3	–	1	–	–	–	–	–	1
Cloning	Spain	Artes	#1	–	1	–	–	–	–	–	1
			#2	–	–	–	–	–	–	1	1
			#3	–	–	–	–	–	–	1	1
Cloning	USA	Lenore	#1	–	–	0.8	–	0.2	–	–	1
			#2	–	1	–	–	–	–	–	1
			#3	–	1	–	–	–	–	–	1
			#4	–	1	–	–	–	–	–	1
Cloning	Canada	Vaughan	#1	–	1	–	–	–	–	–	1
			#2	–	1	–	–	–	–	–	1
			#3	–	1	–	–	–	–	–	1
			#4	–	1	–	–	–	–	–	1
		Vernon	#1	–	1	–	–	–	–	–	1
			#2	–	0.75	–	–	–	0.25	–	1
			#3	–	1	–	–	–	–	–	1
			#4	–	1	–	–	–	–	–	1
			Total	1	23.75	0.8	3	0.2	0.25	10	39

Fig. S1

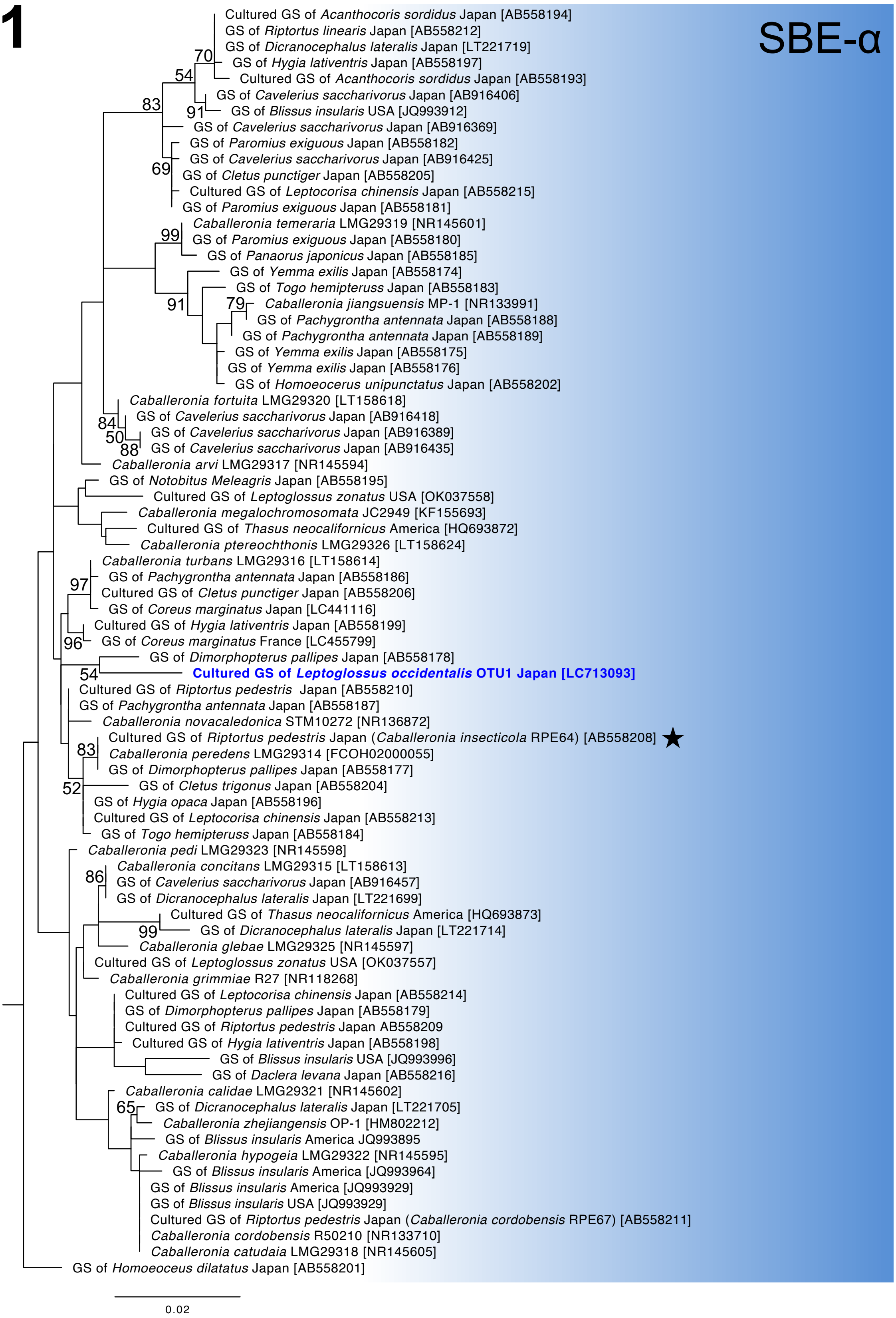

Fig. S1 Uncompressed tree of the SBE-α clade shown in Fig. 2. Several sequences derived from American coreoid insects were excluded from this tree due to a short available sequence length (<1,000 bp), but these sequences were confirmed as SBE-α in the previous phylogenetic analysis (Garcia *et al.,* 2014). Numbers at the tree nodes indicate the maximum-likelihood bootstrap values (%) with 1,000 replicates, and bootstrap values of over 50 are shown. *L. occidentalis* gut symbionts are shown in blue color with bold case. Star: bacterial strain used for symbiont inoculation test in this study. GS: Gut symbiont.

Fig. S2

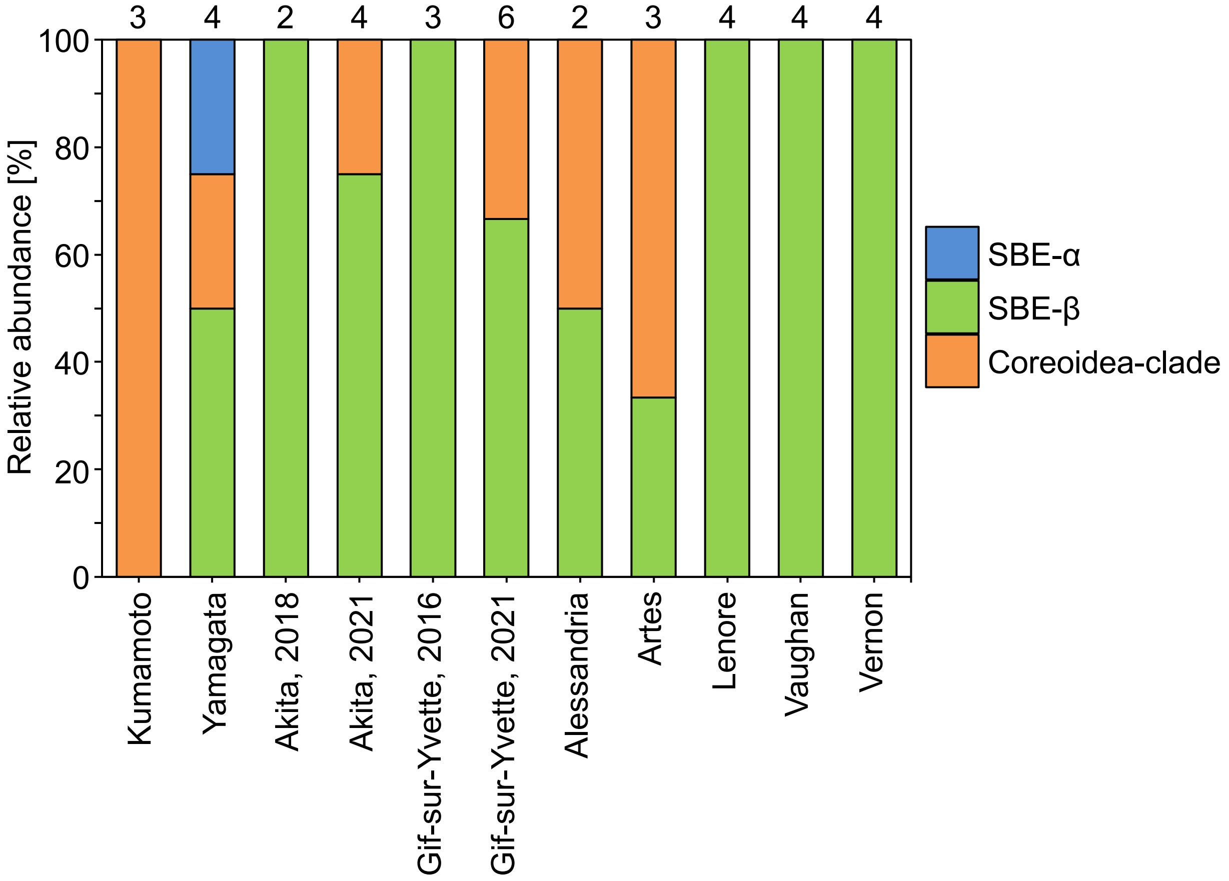

Fig. S2 Relative abundance of SBE-α, SBE-β, and Coreoidea-clade bacteria among gut symbionts of conifer bugs normalized by one OTU per one individual at local level. Number of investigated insects in each city is shown on the graphs, and the precise numbers are provided in Table S1 and Table S2.

Fig. S3

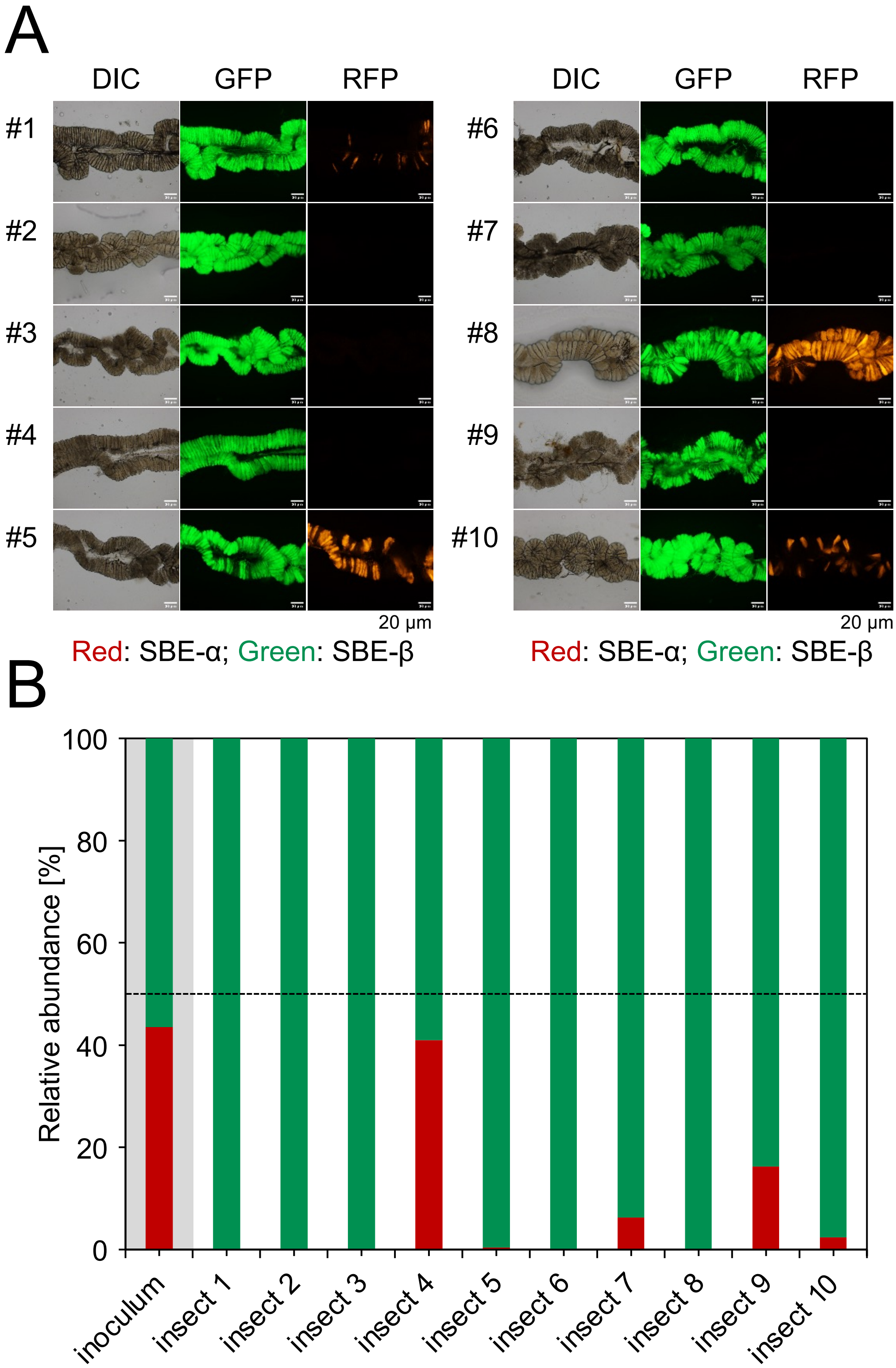

Fig. S3 Competition assay of RFP-labeled SBE-α and GFP-labeled SBE-β strains in the midgut crypts of *L. occidentalis*. (A) Differential interference contrast (DIC) and fluorescence microscopy (GFP and RFP) images of the midgut crypts in 10 individual insects at 7 dpi. Merged GFP and RFP image of the midgut crypts #1 is used in Fig.5A. (B) Relative abundance, determined by flow cytometry, of GFP and RFP strains in the inoculum and in the midgut crypts of 10 individuals at 7dpi. Note that the individuals analyzed in panel B are different from those in panel A.

Fig. S4

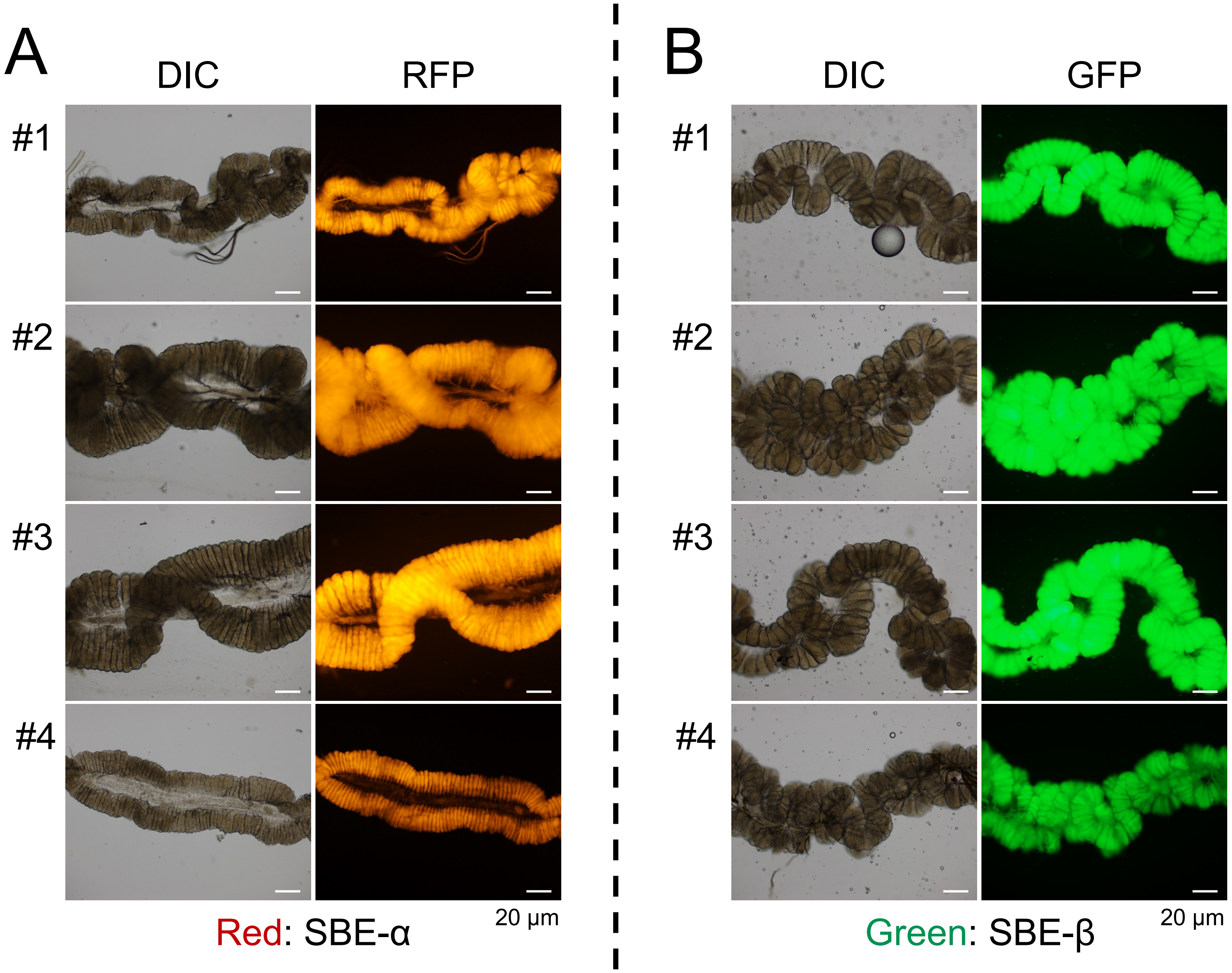

Fig. S4 Single infection of (A) RFP-labeled SBE-α and (B) GFP-labeled SBE-β strains in the midgut crypts of *L. occidentalis*. DIC and fluorescent microscopic (RFP or RFP) images of the midgut crypts in each 4 individual midgut crypts at 7dpi.
